# Severe-malaria infection and its outcomes among pregnant women in Burkina Faso health-districts: Hierarchical Bayesian space-time models applied to routinely-collected data from 2013 to 2018

**DOI:** 10.1016/j.sste.2020.100333

**Published:** 2020-02-15

**Authors:** Toussaint Rouamba, Sekou Samadoulougou, Halidou Tinto, Victor A. Alegana, Fati Kirakoya–Samadoulougou

**Affiliations:** a Clinical Research Unit of Nanoro, Institut de Recherche en Sciences de la Santé, 42, Avenue Kumda-Yonre, Centre National de la Recherche Scientifique et Technologique, 11 BP 218 Ouaga CMS 11, Ouagadougou, Burkina Faso; b Centre d’Epidémiologie, Biostatistique et Recherche Clinique, Ecole de Santé Publique, Université Libre de Bruxelles (ULB), Route de Lennik, 808 B-1070, Bruxelles, Belgique; c Evaluation Platform on Obesity Prevention, Quebec Heart and Lung Institute Research Center, Quebec City, QC G1V 4G5, Canada; d Population Health Theme, Kenya Medical Research Institute-Wellcome Trust Research Programme, Nairobi, Kenya; e Geography and Environmental Science, University of Southampton, Southampton SO17 1BJ, UK; f Centre for Research on Planning and Development (CRAD), Laval University, Quebec, G1V 0A6, Canada

**Keywords:** Severe-malaria, Deaths, Pregnancy, Readiness, Spatio-temporal, Burkina-Faso

## Abstract

Fine-scale hotspots detection is crucial for optimum delivery of essential health-services for reducing severe malaria in pregnancy (MiP) and death cases in Burkina Faso. This study used hierarchical-Bayesian Spatio-temporal modeling to explore space-time patterns and pinpoint health-districts with an exceedance probability of severe MiP incidence and fatality rate. Study also assessed effect of health-district service delivery (readiness) on severe-MiP outcomes.

Severe-MiP fatality rate declined considerably while its incidence rate remained unchanged between January-2013 and December-2018. Severe-MiP cases persisted throughout the year with peaks between August and November. These peaks increased 2.5-fold the fatality rate. Furthermore, severe-MiP fatality was higher in health-districts classified as low-readiness (IRR = 2.469, 95%CrI: 1.632–3.738). However, the fatality rate decreased significantly with proper coverage with three doses for intermittent-preventive-treatment with sulphadoxine-pyrimethamine. Severe-MiP burden was heterogeneous spatially and temporally. The study suggested that health-programs should increase health-districts readiness and optimize resource allocation in high burden areas and months.

## 1 Background

In Sub Saharan African (SSA) countries, malaria infection by *Plamodium falciparum (P.f)* is responsible for most malaria-related deaths leading an estimated number of 404,550 deaths in 2017 (this number represents 93% of 435,000 deaths report by World Health Organization, 2018) globally. Approximately six percent of these global malaria deaths occurred in Burkina Faso in 2018. Children aged under 5 years and pregnant women are the most vulnerable groups (World Health Organization, 2018; Tiono et al., 2014; Ministère de la Santé: Direction Générale des Etudes et des Statistiques Sectorielles, 2018). Apart from the fact that pregnant women are more likely to be affected by severe-malaria due mainly to immunodeficiency induced by pregnancy, malaria puts also the fetus at risk. Indeed, malaria in pregnancy (MiP) is associated with anemia, stillbirth, low birth weight, and maternal and fetal death (Rogerson et al., 2018; Huynh et al., 2015; Desai et al., 2007).

Over the past decade, Burkina Faso government and its international partners have undertaken several health programs in the mindset to reduce the burden of MiP as part of antenatal care (ANC) (Ministère de la Santé 2016). These programs include prompt diagnosis of MiP using the Rapid Diagnostic Tests (RDTs), providing and use of long-lasting insecticidal nets (LLINs), intermittent preventive treatment in pregnancy (IPTp) with sulphadoxine-pyrimethamine (SP) (Ministère de la Santé 2016; Ministère de la Santé/Programme National de Lutte contre le Paludisme 2017). These programs have substantially reduced the case-fatality rate nationally, but seem to have no effect on the reduction of the incidence of severe MiP. Indeed, the case-fatality has decreased from 0.3% to 0.04% nationally between 2013 and 2017, respectively, (Ministère de la Santé: Direction Générale des Etudes et des Statis-tiques Sectorielles, 2018; Ministère de la Santé : Direction Générale des Etudes et des Statistiques Sectorielles, 2014), whereas the reported incidence of severe-MiP increased from 34 to 42 per 1000 pregnant women in the same period.

It is necessary and crucial to develop innovative methods with robust strategies to support the firm commitment of local government and its partners to lead and continue preventing deaths or MiP cases (World Health Organization, 2018). This present study focus on pillar 3 of WHO Global Malaria Program by transforming routinely-collected data on malaria surveillance during pregnancy into essential information for a core intervention and identifying areas that are most affected by malaria burden (World Health Organization, 2018; World Health Organization, 2015). The electronic capture of malaria surveillance data through the District Health Information System (DHIS2) provides an opportunity to inform evidence on MiP spatio-temporal trends and monitor impact of interventions.

For instance in Burkina Faso, the few recent studies that applied advance spatio-temporal statistical modeling methods to malaria data have focused on children less than 5 years or in general population (Ouédraogo et al., 2018a; Ouédraogo et al., 2018b; Rouamba et al., 2019; Diboulo et al., 2015). To our knowledge, no studies to date have focused on pregnant women in Burkina Faso. The aim of this present study was to examine the change of severe MiP using Burkina Faso routine monthly data captured on DHIS2 at health-district level, then explore spatio-temporal pattern of mortality due severe MiP. The study aims also to identify health-districts with exceedance probability of relative risk of death after taken into account the effect of readiness of health-districts to manage malaria cases, seasons, age structure of population of women.

## 2 Methods

### 2.1 Country profile

Burkina Faso is a landlocked Sahelian country (surrounded by six countries) located in the center of West-Africa and covers an area of approximately 272,960 km^2^. The total resident population was estimated at about 20,244,079 inhabitants, the female population represents 51.2% in 2018 and the expected pregnancies per year represent 5.49% of the total population. Globally, the country is characterized by sudanese-type climate, with a long dry season (November to May) and a short-wet season (June to October). In 2018, there were 70 health-districts areas and malaria represented the most common cause of outpatient attendance (43.5%) in peripheral health facilities. There are three species of plasmodium, *P.f* (in more than 90% of cases, responsible for the severe and fatal forms of malaria), *P. malariae* and *P. ovale*. Malaria transmission is stable throughout the country, with a seasonal peak during period from July to October. The planning of control interventions is uniform throughout the country. Currently, IPTp with SP, provision of LLINs and daily ferrous sulfate (200 mg) and folic acid (0.25 mg) are the national policy to prevent MiP and anemia in pregnancy (Ministère de la Santé/Programme National de Lutte contre le Paludisme, 2017; World Health Organization, 2015). Public health facilities provide comprehensive medical care free-of-charge to pregnant women since 2016.

### 2.2 Data sources

The data were drawn mainly from two sources. Data related to malaria (severe cases and deaths) and confounders were downloaded from DHIS2 (https://burkina.dhis2.org). The second source of the data comes from the service availability and readiness assessment (SARA) surveys. These surveys were carried out by the Ministry of Health periodically (every two years) to monitor and assess the availability and readiness of health facilities to provide quality health services.

#### 2.2.1 Data on severe malaria cases and data on deaths due to severe malaria in pregnancy

Data on severe malaria cases and deaths due to severe MiP of the National Malaria Control Program (NMCP) covering the period of January 2013 to December 2018 were used in this study. The data encompassed all pregnant women regardless of their age and attending health facilities (public and private) in whom severe malaria episode or death due to severe malaria was diagnosed and reported. These data (malaria cases and deaths) relied on the monthly reports provided by all health facilities and are an integral part of the Burkina Faso national system for capturing routine health facility data. Data were checked, validated and then captured in DHIS2 by the staff of District Health Information and Disease Surveillance Centers, Hospital Planning and Information Services, and Medical Information Services.

For our analysis, monthly reported severe malaria cases among pregnant women aggregated at health-district level were downloaded from Burkina Faso DHIS2. Likewise, monthly cumulative number of reported deaths due to severe-MiP aggregated at health-district level was also downloaded from DHIS2.

#### 2.2.2 Data on potential confounder variables

Potential confounder data (aggregated by month and by health-district) for the same period (January 2013 to December 2018) were also downloaded from DHIS2 and included proportion of pregnant women who received at least three doses of IPTp with SP and proportion of pregnant women who lived less than 5 km from health-district headquarters. Moreover, the proportion of women under 20 years of age aggregated by year and by health-district was used and was obtained from the projections of Burkina Faso National Institute of Statistics and Demography.

Climate variables for the same period likely to affect malaria transmission through their impact on vector populations, such as monthly average temperature and monthly cumulative rainfall data for each health-district were downloaded from the Global Climate Data website (www.worldclim.org) with 1 km spatial resolution.

#### 2.2.3 Service availability and malaria readiness indicators data

These data on SARA come from two different health facility-based cross-sectional surveys carried-out on 2012 and 2014. These surveys included both private and public health facilities considering the three levels of health system organization (Central, Intermediate and Peripheral) and location (Rural or Urban). In Burkina Faso, SARA survey was designed to have estimates at regional level. The design of SARA survey is reported elsewhere (Ministère de la Santé, 2014).

General service availability such as basic equipment (Adult scale, Thermometer, Blood pressure apparatus and Examination gloves), emergency service (Epinephrine, Atropine and Ambulance), malaria-specific readiness (Malaria diagnostic tests, malaria drugs) and other indicators (Hemoglobinometer, Glucometer, Paracetamol and Ferrous Sulfate + folic acid) were used. Indicators from SARA survey were available as binary variables. If the tracer item was available at the health facility, then indicator is assigned to 1 and 0 otherwise (Ssempiira et al., 2018; Health Statistics and Information Systems, 2015).

### 2.3 Statistical analysis

#### 2.3.1 Analysis of severe MiP incidence rates

*Crude incidence of severe MiP:* To take into account the excess number of severe-MiP cases in a given health-district *k* at a time *t*, standardized incidence ratio (SIR for short) was used to estimate the crude incidence of severe-MiP. Monthly SIR of severe-MiP for each health-district was computed using the following formula: *SIR_kt_* = *Z_kt_/E_kt_*; where *Z_kt_* is the observed number of severe-MiP and *E_kt_* the expected number of severe-MiP in health-district *k* at a month *t*.

The expected number of severe-MiP was computed as *E_kt_* = *α* × *P_kt_*; where *P _kt_* is the total number of pregnant women at risk in a health district *k* at time t and α the overall incidence ratio equal to $Z_{+}/P_{+};(Z_{+}=\sum_{1}^{70}Z_{kt}andP_{+}=\sum_{1}^{70}P_{k})$.

The Mann-Kendall Trend test was used to explore the severe MiP incidence over time, to detect sustained increasing or decreasing trends. The periodicities and seasonality of severe MiP incidence were also assessed using both autocorrelograms and multiplicative decompositions. Furthermore, to better characterize the changes and to detect points of significant changes in severe MiP time-series, change points analysis (Pruned Exact Linear Time as algorithm and Modified Bayesian Information Criterion as penalty) was performed. Then, the time between change points were used to define periods with a high and low incidence of severe MiP.

*Bayesian spatio-temporal analysis of severe MiP incidence rates:* A hierarchical spatio-temporal negative binomial model implemented in a Bayesian framework was fitted to severe MiP cases (*Z _kt_*) to determine the spatial and temporal changes.

$$Z_{kt}∼NegBin\left(\omega_{kt},\tau \right)\text{ where }\omega_{kt}=\tau /\left(\tau +\mu_{kt}\right)$$

In the above formula *τ* is the dispersion parameter and *μ_kt_* is the average monthly number of severe MiP cases in the health-district *k* . The model is formulated as follows:

$$\ln(\mu_{kt})=X_{kt}^{T}\beta +O_{kt}+u_{k}+v_{k}+\gamma_{t}+\phi_{t}+\delta_{kt}$$

where *X_kt_* are space-time covariates associated with the mean process *μ_kt_* with *β* coefficients, *O _kt_* vector of known offset (expected number of severe MiP incidence rates in a health-district k at time t), *u_k_* and *v _k_* is spatially structured risk effect and spatially unstructured risk effect for a specific health-district, respectively, while *γ_t_* and *φ_t_* is temporally structured risk effect and temporally unstructured risk effect for specific month, respectively. *δ_kt_* is the term of interaction between space and time random effects.

Space-time heterogeneity of severe MiP was considered via overall month random effect (structured and unstructured), overall spatially random effect (structured and unstructured). The structured random effects were fitted via prior random walk of order 2 for the time (*γ*_*t*_) and conditional autoregressive (CAR) for the space (*u*_k_). While the unstructured random effects were fitted via prior Gaussian distributions with non-informative parameters for both time (*ϕ_t_*) and space (*v_t_*). A space-time interaction was included and fitted using prior Gaussian distribution. Based on the smallest deviance criteria (DIC) (Spiegelhalter et al., 2002), the type I space-time interaction which interact the unstructured temporal effects and spatially unstructured effects was chosen. The spatial autocorrelation was captured through a binary neighborhood matrix *W* (70 × 70 symmetric matrix) based on borders sharing; if two health-districts *k* and *j* share a common boundary, *w_kj_* is assigned to 1 and 0 otherwise.

Then, the model was adjusted by covariates related to health-district composite readiness profile, seasons of malaria transmission, temperature, rainfall, proportion of women who received at least three doses of IPTp with SP, proportion of women living less than 5 Km to health-district headquarters, proportion of women under 20 years (to account for high risk of malaria in primi-gravida). Comparisons of the two models (with and without covariates) were performed using the DIC. After taking into account the covariate effects, the conditional predictive ordinate (CPO) and Cramer-von Mises test of goodness-of-fit (Null hypothesis: “*uniform distribution*”) was used to check the internal validation of the models (Czado et al., 2009; Schrödle and Held, 2011). The posterior exceedance probability $\left(\hat{\mathrm{Pr}}(\xi_{k}>c)>b\right)$ of exceeding 23 cases of severe malaria per 1000 pregnant women in 2018 was computed to identify health districts with excess risk. This threshold was derived from the objective of the NMCP that aims to reduce malaria incidence rate globally at least 40% by 2020 compared with 2015.

#### 2.3.2 Analysis of case fatality

*Crude case fatality:* Crude severe-malaria fatality rates (SMFR) for each month and each health-district were computed from the observed number of deaths (*Y_k t_*) and observed number of severe-MiP (*Z_kt_*) in health-district *k* at a month *t* using the following formula: *SMFR _kt_* = *Y_kt_/Z _kt_*.

Like was done for the severe MiP incidence above, the temporal trend and seasonality of SMFR was assessed through Mann-Kendall Trend test and autocorrelograms/multiplicative decompositions, respectively.

*Bayesian analysis of case fatality:* In this study, since there is overdispersion caused by an excessive number of zeros, Zero-Inflated Poisson (ZIP) model was suitable to handle the data (Alegana et al., 2013; Lambert, 1992; Bohning, 1998). Thus, a spatio-temporal Zero-Inflated Poisson model implemented in a Bayesian framework was fitted to number of maternal deaths to determine the spatial pattern, temporal trend and space-time interaction of SMFR. For Zero-Inflated Poisson model, probability of observing zero is:

$$P_{kt}(Y_{kt}=0)=\;\;(1-\pi_{kt})+\pi_{kt}e^{-\theta_{kt}}0\leq p<1$$

$$\begin{array}{cc} P_{kt}(Y_{kt}=\text{λ})=\pi_{kt}\frac{\theta_{kt}^{\text{λ}}}{\text{λ!}}e^{-\theta_{kt}} & \text{λ}=1,\;\ldots ,\;\;\infty \end{array}$$

In the equation above, (1 – *π_kt_*) represents the probability of observing a true zero and, therefore, when *π* = 1 the equation is reduced to a general Poisson model and zero is inflated when *π* < 1. The spatio-temporal distribution of observed death cases was modelled as:

$$Y_{kt}∼ZIP(\pi_{kt}\theta_{kt})$$

$$\ln(\theta_{kt})=X_{kt}^{T}\beta +O_{kt}+\eta_{kt}$$

$$\eta_{kt}=u_{k}+v_{k}+\gamma_{t}+\phi_{t}+\delta_{kt}$$

where, *θ_kt_* is the expectation (mean) of observed death case count *Y_kt_*, *X_kt_* are explanatory space-time covariates associated with the mean process *θ_kt_* with βcoefficients, *O_kt_* a space-time vector of known offset (expected number of maternal deaths in a health-district k at time t) and *η_kt_* is spatio-temporally autocorrelated random effects with space-time interaction.

The spatial autocorrelation of SMFR was considered through two components of spatially random effects: structured (*u_k_*) and unstructured (v*k*) (Besag et al., 1991). They were fitted via prior a CAR distribution for *v_k_*, and prior Gaussian distribution with non-informative hyperparameters for *Vk* (Banerjee and Fuentes, 2012). Like the bayesian spatio-temporal modeling of severe MiP incidence rates, the spatial autocorrelation in this model was captured via a binary neighborhood matrix.

The temporal correlation over the 72 months (six years) was captured in the model using a Gaussian distribution with non-informative hyperparameters for *φ_t_* and random walk of order 2 for *γ _t_* .

For interaction (*δ_kt_*), several null models with space-time interaction were examined. Then, the interaction between *v_k_* and *ϕ_t_* or type I (*R_δ_* = *R_v_* ⊗ *R_ϕ_* = *I* ⊗ *I* = *I*) was chosen as the suitable type of interaction for capturing the space-time dynamic of SMFR between the health-districts (Knorr-Held, 2000).

The retained null model with interaction was adjusted by covariates related to health-district composite readiness profile, seasons of malaria transmission, proportion of women who received at least three doses of IPTp with SP, proportion of women living less than 5 Km to health-district headquarters, proportion of women under 20 years. Comparisons of the two models (with and without covariates) were performed using the DIC. After taking into account the covariate effects, the conditional predictive ordinate (CPO) and Cramer-von Mises test of goodness-of-fit (Null hypothesis: "*uniform distribution*") was used to check the internal validity of the models (Czado et al., 2009; Schrödle and Held, 2011).

The posterior exceedance probability $\left(\hat{\mathrm{Pr}}(\xi_{k}>c)>b\right)$ of SMFR according to a threshold (c) was computed and mapped for each health-district. Here, b represents the risk categorization according to Richardson; in this categorization a health-district is considered as high risk if $\hat{\mathrm{Pr}}(\xi_{i}>c)>0\cdot 8$ (Richardson et al., 2004). In this study, probability of exceeding thresholds (0.19% in 2018) according to the NMCP target was also assessed.

In this study, missing values in covariates were addressed by mean imputation. A missing value for given health-district (*k*) at month *t* (*X_kt_*), imputation was performed by imputing mean value obtained from *X*_*kt*-1_ and *X*_*kt*+1_.

To compute the posterior distributions of the marginal random effects and fixed effects of the model, the Integrated Nested Laplace approximation package of R (R-INLA) was used.

#### 2.3.3 Analysis of readiness indicators

Malaria readiness indicators, general and emergency equipment availability indicators for each health-district was estimated using SARA survey data. This was performed firstly, through a hierarchical spatial binomial modeling implemented in a Bayesian framework to estimate predicted posterior mean of proportion of health facilities with the availability of equipment and malaria readiness tracer by health-district. Secondly, a hierarchical ascending classification (HAC) on the estimates of fitted values of availability and readiness was performed to assess the resemblances and differences between health-districts from a multidimensional point of view. For the HAC, Euclidean distance and Ward’s criterion were used. The HAC algorithm allows to have different groups of health-districts by minimizing the total within-cluster variance and maximizing the total between-cluster variance.

## 3 Results

### 3.1 Summary of crude estimates incidence and deaths

**Figure 1:**
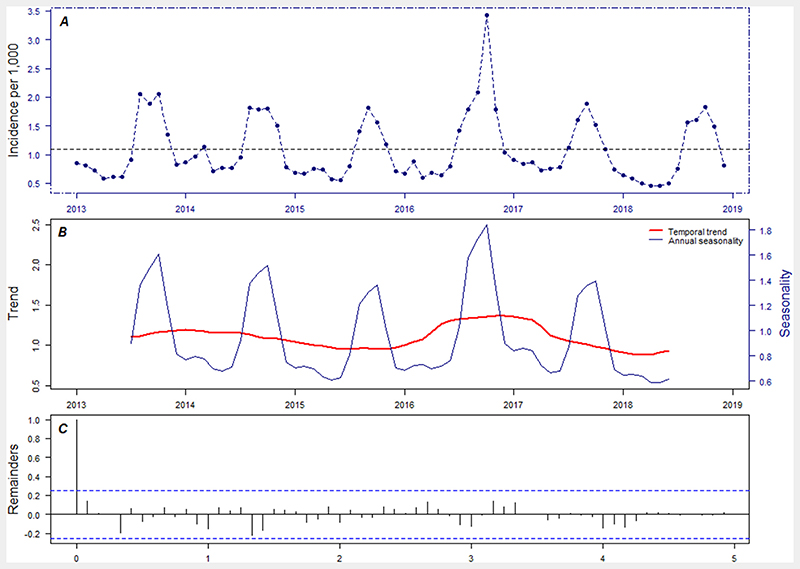
Monthly severe-malaria incidence among pregnant women from 2013 to 2018. (A) temporal pattern in the original time series (reported cases). (B) Decomposed components into seasonal and trend component. (C) Decomposed remainder component.

From the observed data, the incidence of severe MiP in Burkina Faso during the study period was constant (there was no sustained increasing or decreasing trend). Then, there were annual seasonal fluctuations in the incidence of severe MiP (Fig. 1). This seasonality was uniformly distributed annually with two seasons or periods each year (see Fig. S1 in Supplementary Information file). One season where the incidence of severe MiP was high (between August and November) and one season where the incidence of MiP was low (outside the interval of August and November).

**Figure 2:**
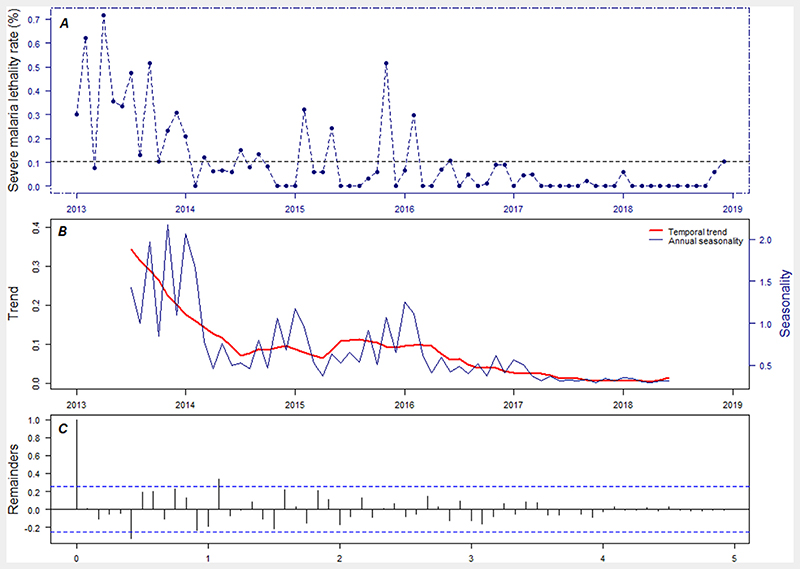
Severe-malaria fatality rate among pregnant women from 2013 to 2018. (A) temporal distribution. (B) Decomposed components into seasonal and trend component. (C) Decomposed remainder component.

There was a total of 171,479 severe-MiP cases and 156 deaths among pregnant women during the study period. Regarding the severe-malaria related deaths in pregnancy, the findings showed a sustained decreasing trend in SMFR. Indeed, the SMFR decreased from 0.3 in 2013 to 0.02 per 100 pregnant women in 2018 (Fig. 2). In addition when analyzing, the geographic distribution of SMFR, there was a heterogeneity across the country, where the location of areas with high fatality rates varied according to the periods of severe malaria transmission. Health-districts of Solenzo, Orodara and N’Dorola exhibited high values of SMFR during the season of the high incidence of severe-malaria, whilst health-districts of Gayeri, Boulsa and Batié exhibited the highest value of SMFR during the period of the low incidence of severe malaria (see Fig. S2 in Supplementary Information file).

### 3.2 Malaria service readiness from SARA surveys

Table 1 summarizes the composite readiness profile of health-districts for managing malaria cases. Three profiles of composite readiness among the 70 health-districts were found (See Fig. S3 in Supplementary Information file). According to the percentage of availability of general service and malaria-specific item tracers, these profiles were described as Low (*n* = 6), Medium (*n* = 20) and High (*n* = 44) readiness profiles. Compared to Low and Medium profile, the High readiness profile was characterized mainly by a high rate of availability of essential drugs, basic equipment, malaria diagnostic capacity and emergency transport.

### 3.3 Bayesian model validation

The comparison of Bayesian model validation based on posterior parameters for both severe-MiP incidence and fatality cases was summarized in Table 2 . The covariates improved the models fit marginally as indicated by the lower DIC for adjusted models in both severe-MiP incidence and fatality cases modeling.

Regarding the severe MiP incidence modeling, the values of conditional predictive ordinate (CPO) were small and were estimated at 0.030 and 0.008 for the model without and with covariates, respectively, which indicates greater predictive accuracy (Schrödle and Held, 2011). Moreover, this also suggests a big difference between the two models without and with covariates. Likewise, for the case fatality modeling, the values of CPO were estimated at 0.111 and 0.114 for the model without and with covariates, respectively, and since a smaller CPO value usually indicates greater predictive accuracy. However, this also suggests that there was not a big difference between the two models fitted for case fatality without and with covariates.

### 3.4 Effects of covariates

The effect of covariates on severe MiP and case fatality as well as random effects were summarized in Table 3. The results showed that low readiness score (IRR = 1.503, 95% CrI: 1.106; 2.024) and medium readiness score (IRR = 1.476, 95% CrI: 1.331; 1.637) had significant association with incidence of severe MiP by multiplying the risk at about 1.5-fold. In addition, the high malaria transmission period produced a positive and significant association with severe MiP incidence (IRR = 2.138, 95% CrI:1.970; 2.323). However, the incidence of severe MiP decreased significantly with the increase in the proportion of women receiving at least three doses of IPTp (IRR = 0.902, 95% CrI: 0.864; 0.996). Likewise, severe MiP incidence decreased significantly with the increase in average temperature (IRR = 0.997 95% CrI: 0.996; 0.998).

Health-district readiness and transmission periods of severe MiP exhibited a statistically effect on SMFR. For readiness profile, severe MiP fatality increased in case of low readiness of health-district (IRR = 2.458, 95% CrI: 1.652; 12.096). Findings showed that severe MiP fatality was statistically higher during high incidence period of severe-malaria (IRR = 2.469, 95% CrI: 1.632; 3.738). Additionally, findings showed a reduction in the number of severe-MiP fatality when the proportion of women receiving at least three doses of IPTp and women living less than 5 Km to health-district headquarters increased, but these associations were not statistically significant.

### 3.5 Bayesian results for the analysis of incidence and case fatality

The posterior overall temporal trend of severe MiP from null model showed a constant trend, albeit the incidence peaked globally in the months of July to October and decreased in the month of February to May each year. However, after taking into account for the covariate effects, there was a slight increase in temporal trend between July 2016 and January 2017 and a slight decrease between February 2018 and May 2018 (Fig. S4 in Supplementary Information file).

There was a strong spatial dependency in severe MiP incidence as suggested by the spatial fractional variance value (Table 3). The results showed a heterogeneity across the country (Fig. S5 in Supplementary Information file). The adjusted model for severe-MiP identified 30 health-districts which displayed a probability of exceeding the NMCP threshold (23 cases per 1000 pregnant women) estimated for 2018. The geographical distribution of this excess of risk was heterogeneous across the country (Fig. S6 in Supplementary Information file).

**Figure 3:**
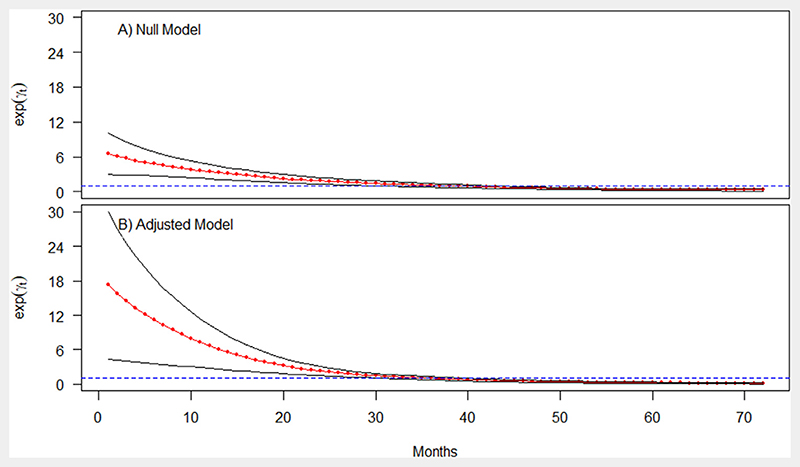
Posterior temporal trend of structured effects of malaria related death among pregnant women in Burkina Faso health-districts. The red bold line describes the posterior mean of temporal trend of unstructured effect and the black line the 95% credibility interval.

Regarding the temporal trend (or temporal relative risk) of case fatality, the results showed a significant decreasing of risk of death between January 2013 and June 2014. From July 2014, a slow reduction of risk of death due to severe MiP was observed. Between July 2014 and August 2016, the reduction in the risk of death due to severe MiP was not significant. However, after August 2016 the reduction in the risk of death due to severe MiP became significant (Fig. 3).

**Figure 4:**
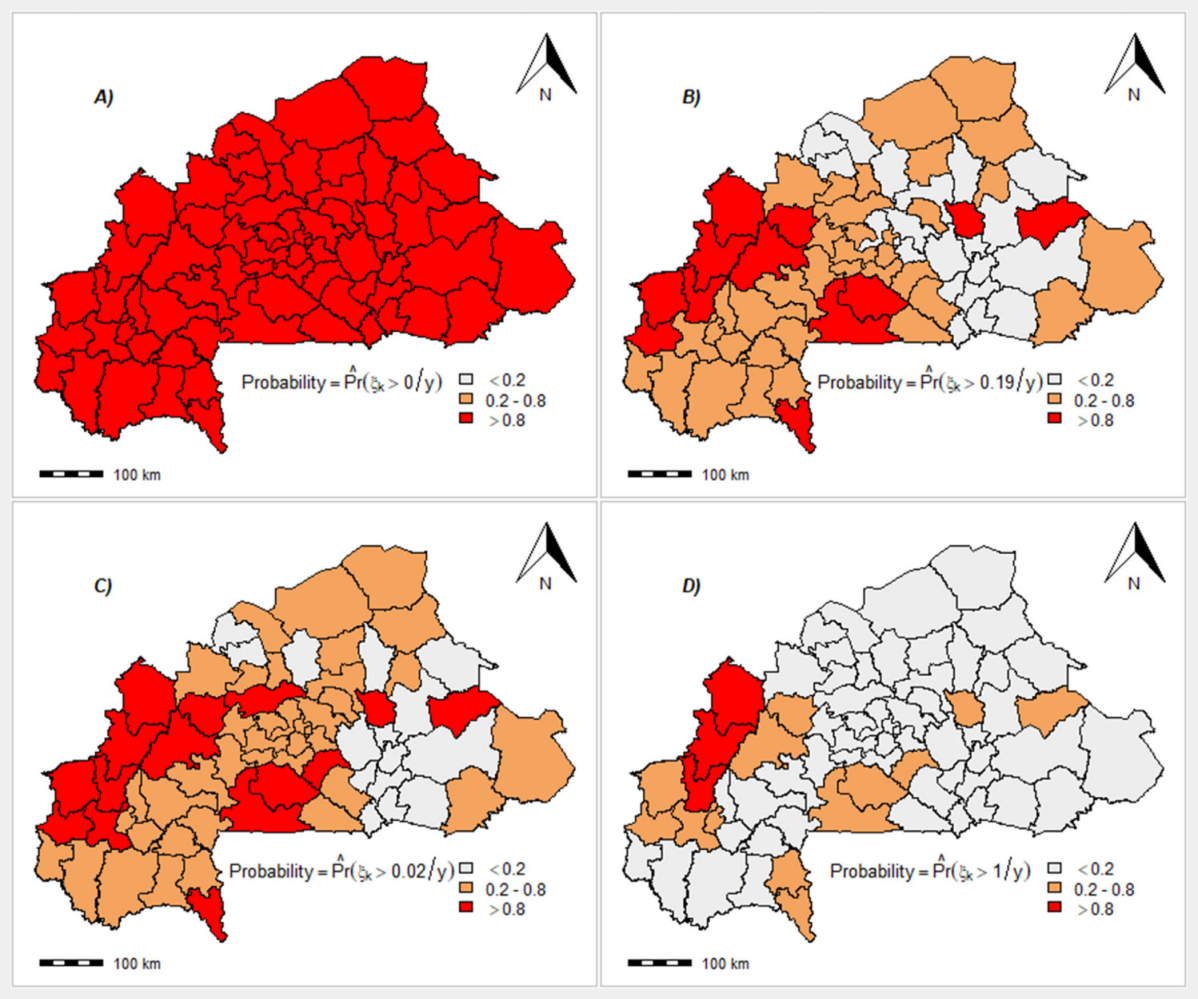
Burkina Faso health-districts severe-malaria deaths among pregnant women: exceedance probability $\left(\hat{\mathrm{Pr}}(\xi_{k}>c/y\right)$ of posterior expected relative risk basedasedbased. Top left, A: *c* = 0 (No death); Top right, B: *c* = 0.19 (NMCP threshold in 2018); Bottom left, C: *c* = 0.02 (fatality rate from raw data in 2018); Bottom right, D: *c* = 1 (Richardson threshold). The threshold of 0.19% is derived from the NMCP strategy plan that aims to reduce the global annual average rate of mortality rate due to malaria by 2 . 4 per 100,000 for the period 2015–2020 (Ministère de la Santé 2016). The level of SMFR in pregnant women was estimated at 0 . 24% in 2015 (Ministère de la Santé: Direction Générale des Etudes et des Statistiques Sectorielles, 2018).

For the spatial pattern of risk of death due to severe MiP, results showed a heterogeneity across the country. The spatial fractional variance showed a high value (98.3% and 75.4% for null and adjusted model, respectively) that indicates a strong spatial dependency. Health-district located mainly in Boucle de Mouhoun, Haut-bassins and Central-west region of the country exhibited the higher risk (Fig. S7 in Supplementary Information file). Furthermore, the spatial distribution of predicted values of the number of deaths exhibited a spatial heterogeneity (Fig. S8 in Supplementary Information file). The adjusted model identified 12 health-districts which displayed a probability of exceeding the NMCP threshold (0.19 death per 100 pregnant women) estimated for 2018 (Fig. 4A). There was four health-districts located in Boucle de Mouhoun region (Nouna, Solenzo, Dédougou and Toma), three in Haut-bassins region (Dandé, Orodara and N’Dorola), two in Central-west region (Léo and Sapouy), one in south-west region (Batié), one in East region (Gayéri) and one in Central-East region (Boulsa). Considering Richardson classification (probability of exceeding one death), three health-districts (Nouna, Solenzo and Dandé) exhibited exceedances of risk (Fig. 4D).

## 4 Discussion

Severe malaria is an important cause of maternal mortality in Burkina Faso (Ministère de la Santé 2016). One of the most important objectives of Burkinabe government is to improve the health of the population by significantly reducing the burden of malaria by 2020. In terms of practical objectives, reduce globally the mortality related to severe-malaria from 29 per 100,000 in 2015 to 17 per 100,000 in 2020. In this study, a Hierarchical Bayesian spatio-temporal approach was applied to routine data aggregated by month and health-district (operational entity of health system) for providing accurate estimates on the burden of MiP in term of severe-malaria related deaths in pregnancy.

Our results show that severe-malaria-related deaths are rapidly declining compared to the target set by the NMCP.

This significant reduction in the fatality rate in Burkina Faso setting is consistent with the decrease recorded in other countries in the sub region of West-Africa (World Health Organization, 2018). By contrast, our study showed while the numbers of deaths was decreasing, the numbers of severe-MiP cases was stable thorough the study period, with peaks during the month of August and the month of November each year. This highlight the scale of problem in achieving SDG3.1 (“reduce the global maternal mortality ratio to less than 70 per 100 000 live births by 2030”) and SDG3.3 (“Ensure healthy lives and promote well-being for all at all ages” by reducing malaria burden in pregnancy).

Several factors could explain this relative stability in the number of cases of severe MiP during the study period. In the majority of SSA countries characterized by poor health system performance, only a small proportion, qualified as “hippopotamus ears”, are diagnosed (clinical and/or biological) and reported by the health system (Breman, 2001). Thus, the implementation of health programs aiming at improving the accessibility and quality of health care, as well as the surveillance system, should provide a reliable overview of the extent of the disease. In Burkina Faso, several maternal health programs have recently been implemented, including the integration of MiP data into DHIS2 (in 2013), results-based financing (in 2014), decentralization of health care through communities’ projects (in 2015), and free of charge of health care among pregnant women (in 2016). These measures would improve health facilities capacity in diagnosing comprehensively malaria cases, (Lechthaler et al., 2019) and effective treatment (resulting in reduction of bad adverse outcomes of diseases i.e., deaths).

Accurate malaria routine data associated with good modeling are crucial for monitoring progress towards the SDG3. Our findings which pinpointed the months and areas where the risk is higher, could be used as relevant tools for strengthening the surveillance and improved related-factors such as strength of health system readiness and available resources.

Our results suggested that severe-MiP-related mortality estimated to be 2.5 times in health-district classified as lower readiness profile (compared to those classified as high readiness) is in agreement with findings from study that measured health facility readiness and its effects on severe-malaria outcomes in Uganda (64% lower in general population health facilities with high readiness). In this later study SARA data were used in multiple correspondence analysis (MCA) to build a composite facility readiness score (Ssempiira et al., 2018). In our study, health-districts belonging to high readiness profile were characterized by a high availability rate of essential drugs, basic equipment, malaria diagnostic capacity mainly RDT and emergency transport. A high readiness level of the health facilities remains a vital component of malaria control strategies, which allow easily an effective malaria case management by early diagnosis and prompt treatment with effective antimalarial drugs (World Health Organization, 2015). Moreover, high readiness profile ensures prompt referral of severe cases of diseases to the appropriate level equipped to provide adequate support services and cares.

The burden level of SMFR was not uniformly distributed across the country. Since Burkina Faso is in a context where resources are very limited, the pinpointed high-risk periods and high-risk areas could be targeted by health policy makers for deploying the necessary interventions effectively and for advocating allocation of resources.

One strength of the study is the use of hierarchical cluster analysis to build the readiness groups (health-districts which share specific properties in common) from data of SARA survey. It is a practical method in categorizing meaningful clusters within data sets that may superficially appear homogeneous (Everitt et al., 2011; Yim and Ramdeen, 2015). Additionally, the interpretation of readiness group is practical and easy and can be used without difficulty by decision makers to improve significantly readiness factors or indicators.

One limitation of this study is the fact that in developing countries, health facility records underestimate most often morbidity and mortality due low attendance rate of health services and home deaths. Thus, it is possible that a part of patients who do not attendance health facilities remain undocumented by the surveillance system and are not captured on DHIS2. However, this issue could be considered as very low in Burkina Faso setting. Indeed, regarding the population of pregnant women, annual health statistic and SARA survey reported a high levels of ANC coverage (83.0%) associated with high level of data promptness and completeness reporting (99.6%) and high level of data quality (Concordance index, 95.5%). In addition, the parasitological confirmation rate of malaria cases in health facilities in 2017 was estimated at 91.7%.

## 5 Conclusion

Routine data from malaria surveillance system are important for tracking spatial and temporal burden of malaria disease and deaths. From 2013 to 2018, Burkina Faso has experienced an exponential decline in severe MiP case fatality, despite a stability of severe-malaria cases. The country is characterized by a spatial and temporal heterogeneity of malaria-related deaths, this has allowed an identification of areas and periods of year where the risk of death was excessive. Higher health-district readiness is associated with a reduced risk of severe-malaria bad outcomes. Thus, in order to sustain this substantial reduction in severe-malaria burden, and in a meantime to achieve the SG3, national malaria control program should optimize the resources allocation in favor to these hotspots. Moreover, since the global funding for malaria has remained relatively stable since 2010, the country should give a greater weight to health in the allocation of government revenues.

## Supplementary Material

Supplementary material associated with this article can be found, in the online version, at 10.1016/j.sste.2020.100333.

Supplementary information

## Figures and Tables

**Table 1:** Characteristics of health-districts obtained by the hierarchical ascendant classification estimated from posterior mean implemented in Bayesian binomial model.

Readiness indicators	Percentage (sd) of available readiness tracer items per group			
	Whole *N* = 70	High *n* = 44	Medium *n* = 20	Low *n* = 6
*Basic equipment*				
Adult scale	96% (0.9)	96% (0.8)	96% (0.7)	95% (0.8)
Thermometer	99% (0.4)	99% (0.3)	99% (0.5)	100% (0.1)
Blood pressure apparatus	90% (7.3)	93% (3.0)	67% (7.1)	89% (3.5)
Examination gloves	87% (11.8)	91% (7.8)	90% (3.4)	81% (15.4)
*Biological diagnostic*				
Malaria RDTs	88% (17.1)	98% (0.8)	92% (4.4)	69% (18.3)
Hemoglobinometer	12% (2.3)	12% (1.6)	16% (3.2)	11% (2.0)
Glucometer	8% (5.3)	8% (2.1)	15% (4.4)	7% (7.9)
*Basic emergency equipment*				
Epinephrine (injection)	12% (1.8)	12% (1.6)	13% (1.4)	12% (2.2)
Atropine (injection)	35% (35.4)	10% (0.7)	12% (1.9)	85% (8.3)
Ambulance	90% (5.1)	93% (2.7)	80% (6.9)	86% (2.7)
*Essential drugs*				
Severe-malaria				
Artesunate (rectal or injection)	61% (42.2)	93% (2.8)	69% (5.3)	2% (0.6)
Uncomplicated malaria				
ACT tablets	90% (7.7)	93% (2.8)	69% (5.3)	90% (7.6)
Malaria prevention				
Sulphadoxine-Pyrimethamine	90% (6.6)	93% (2.8)	69% (5.3)	89% (3.4)
Others essential drugs
Paracetamol	93% (9.8)	96% (2.8)	63% (12.9)	93% (6.1)
Ferrous Sulfate + folic acid	86% (13.4)	92% (3.3)	69% (5.2)	77% (17.6)

RDT: Rapid Diagnostic Tests.

ACT: Artemissinin-based Combinaison Therapies.

**Table 2:** Spatial fractional variance, posterior mean deviance, the DIC, the number of effective parameters and CPO score for each implemented model.

	Severe malaria		Case fatality	
	Null model	Model adjusted^¥^	Null model	Model adjusted^¥^
Spatial fractional variance	62%	79%	98.3%	75.4%
Posterior mean of the deviance ($\bar{D}$)	35390.25	32714.4	1041	66,201
DIC	38007.88	35785.09	108,234	107,071
CPO	0.030	0.008	0.111	0.114
Effective number of parameters (*PD*)	2617.63	3086.923	49.76	46.90

CPO, conditional predictive ordinate; DIC, deviance information criterion.

¥ Adjusted by age structure (Proportion of women less than 20 years).

**Table 3:** Posterior estimates of the effects covariables on severe-malaria and severe-malaria related deaths in pregnancy estimated from Hierarchical Bayesian models.

	Severe malaria Posterior mean, (95% CrI)		Case fatality Posterior mean, (95% CrI)	
	Null model	Model adjusted^¥^	Null model	Model adjusted^¥^
*Fixed effects* ^†^				
Intercept	0.011 (0.010; 0.012)	0.003 (0.001; 0.012)	2.588 (1.564; 4.395)	1.056 (0.038; 2.132)
Readiness				
High	..	1	..	1
Medium	..	1.476 (1.331; 1.637)	..	1.839 (0.817; 4.499)
Low	..	1.503 (1.106; 2.024)	..	2.458 (1.652; 12.096)
Transmission periods				
Low	..	1	..	1
High	..	2.138 (1.970; 2.323)	..	2.469 (1.632; 3.738)
Proportion of women who received at least three doses of IPTp with SP	..	0.902 (0.864; 0.996)	..	0.997 (0.984; 1.001)
Less than 5 km to district headquarters^†^	..	1.002 (0.998; 1.006)	..	0.998 (0.968; 1.030)
Climatic factors				
Rainfall (mm)	..	1.010 (1.000; 1.024)	..	..
Average temperature (°C)	..	0.997 (0.996; 0.998)	..	
*Random effects on a log scale*				
Marginal random effects (Heterogeneity)				
HD specific residuals (*u_k_* + *v_k_*)	0.003 (−0.143; 0.143)	0.005 (−0.178; 0.178)	0.217 (−1.189; 1.168)	0.248 (−1.276; 1.256)
Structured trend random effect (*γ*)	0.006 (−0.114; 1.114)	0.013 (−0.161; 0.161)	0.102 (−0.448; 0.447)	0.156 (−0.533; 0.531)
Unstructured trend random effect (*ϕ*)	0.000 (−0.023; 0.023)	0.010 (−0.150; 0.150)	0.672 (−0.263; 1.251)	0.000 (−0.010; 0.010)
Interaction random effect (*δ*)	−0.002 (−0.541; 0.539)	−0.001 (−0.487; 0.484)	0.000 (−0.025; 0.025)	0.000 (−0.019; 0.019)

* Statistically significant.

CrI, Credible intervals; HD: Health district; IPTp, Intermittent Preventive Treatment in pregnancy; SP, Sulphadoxine-Pyrimethamine.

¥ Adjusted by age structure (Proportion of women less than 20 years).

† IRR: Incidence Rate Ratio.
