# Supplementary material for: Severe-malaria infection and its outcomes among pregnant women in Burkina Faso health-districts: Hierarchical Bayesian space-time models applied to routinely-collected data from 2013 to 2018
Source: Spat Spatiotemporal Epidemiol. Author manuscript; Available in PMC 2022 Sep 6. (PMC7613547; doi:10.1016/j.sste.2020.100333)
Supplement: Supplementary information [file EMS153511-supplement-Supplementary_information.docx]

**Supplementary Information files**


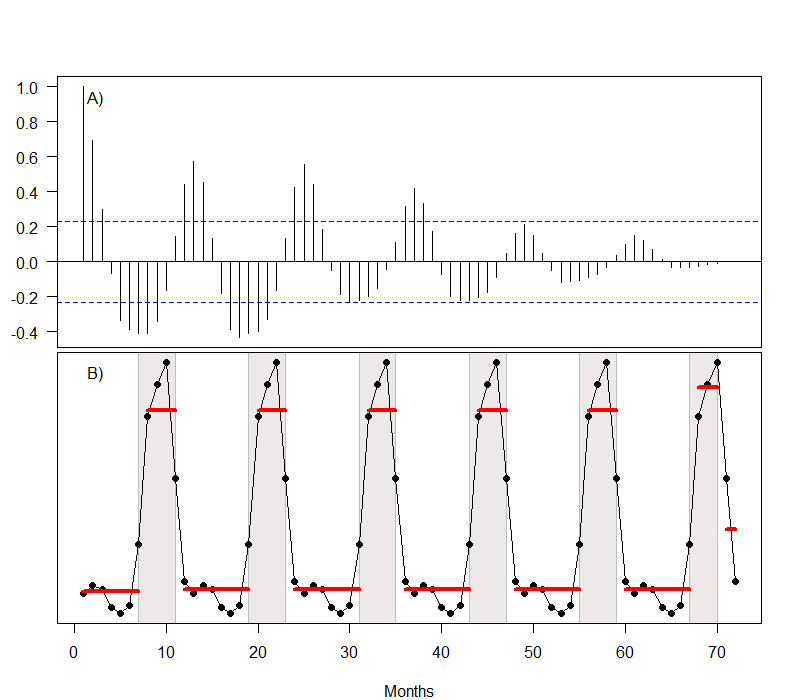


**Figure S1.** Seasonality pattern of severe-malaria incidence in pregnant women

A) Autocorrelogram of monthly severe-malaria incidence. B) Period of intensity of monthly severe-malaria incidence obtained from change point analysis of decomposed components into seasonal. The horizontal red bold lines indicate segment length (no. of observations between changes) or duration of each period.

**
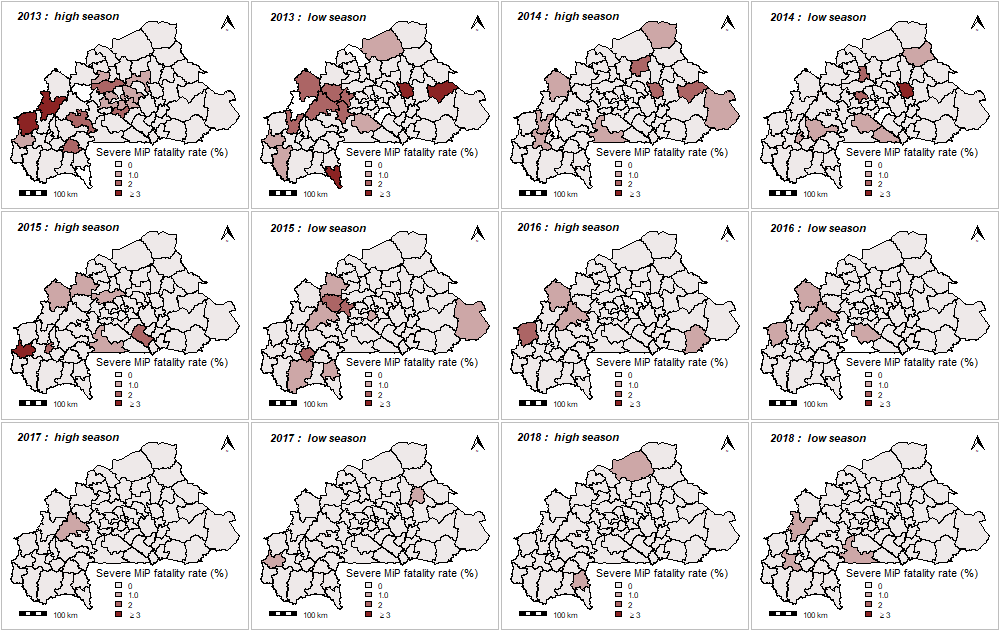
**

**Figure S2.** Geographic distribution of observed monthly severe-malaria fatality among pregnant women in Burkina Faso health-districts from 2013 to 2018

High season, period between July and November of each year. Low season, period between outside the interval of July and November of each year.

Source : The shapefile is obtained from Base Nationale de Découpage du territoire du Burkina Faso (BNDT, 2006). Maps created by Toussaint Rouamba, 2019

**
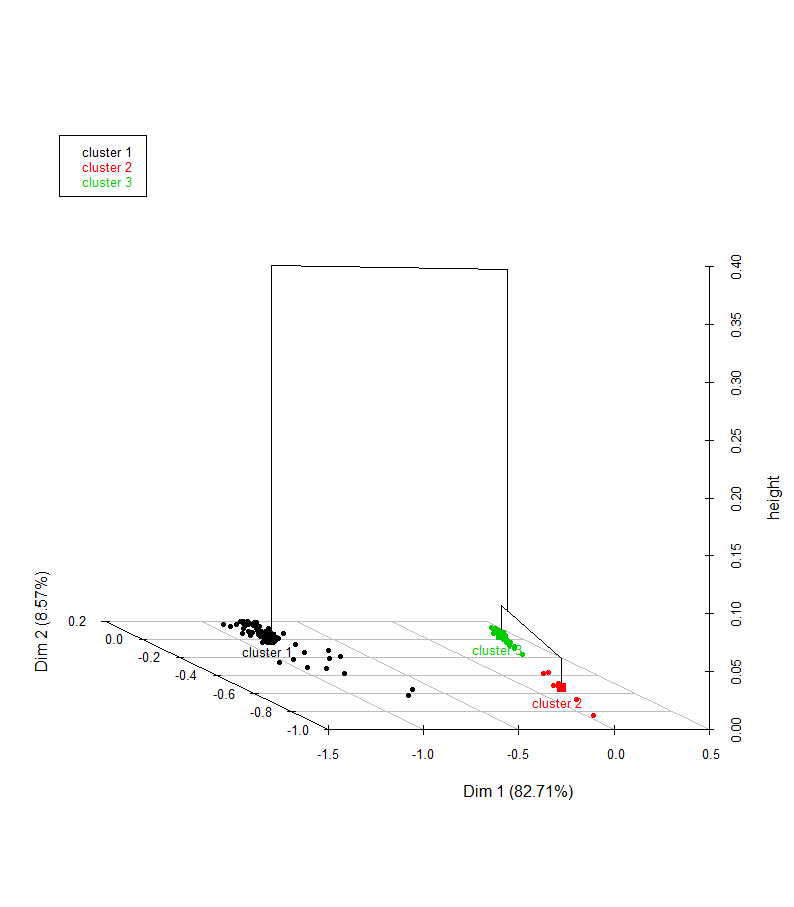
**

**Figure S3.** Representation of the three clusters obtained from the hierarchical ascendant classification estimated from posterior mean implemented in Bayesian binomial model

**
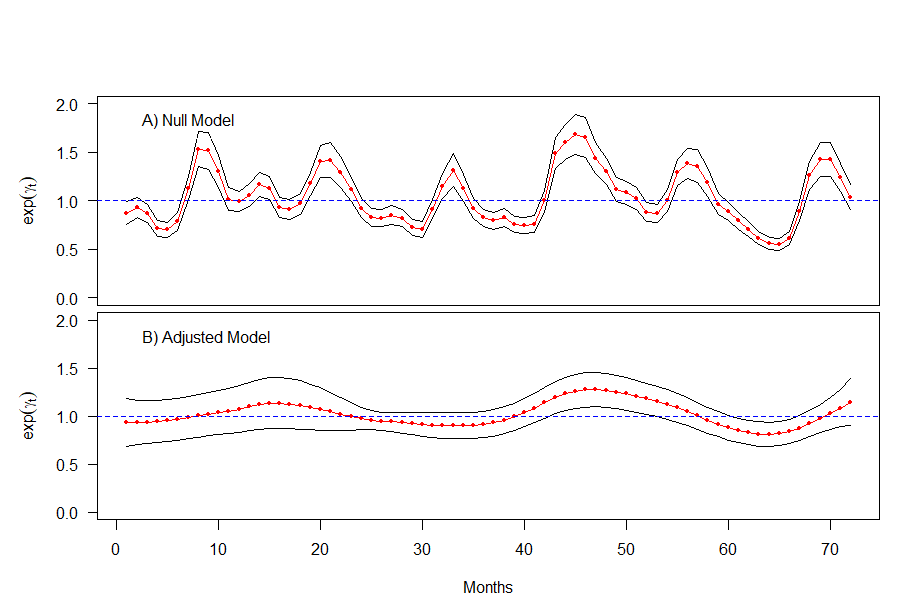
**

**Figure S4.** Posterior temporal trend of structured effects of severe malaria cases among pregnant women in Burkina Faso health-districts

**
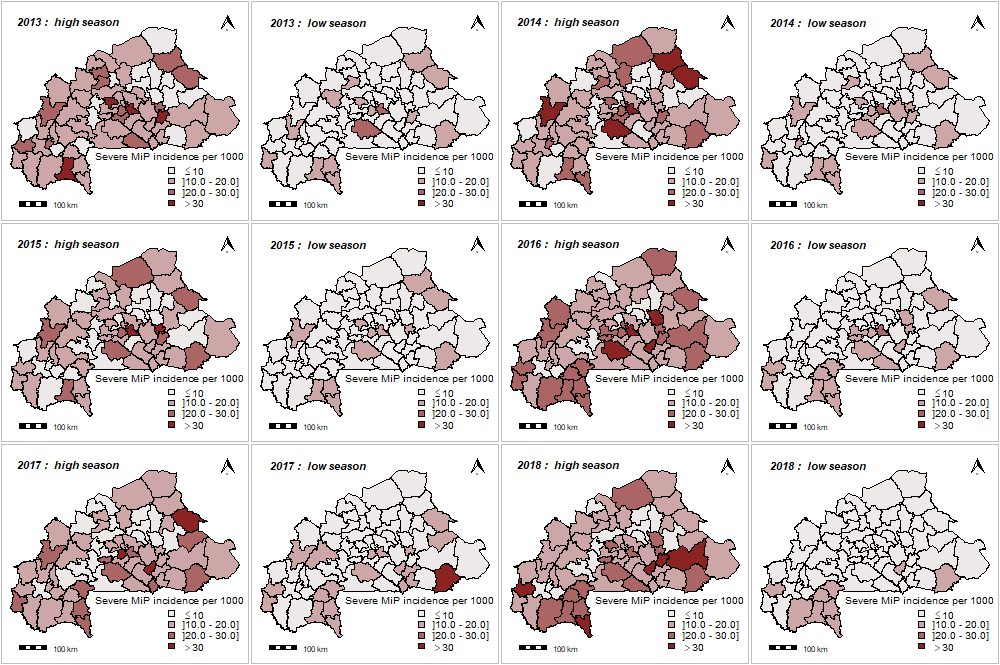
**

**Figure S5.** Geographic distribution of posterior mean of severe-malaria cases among pregnant women in Burkina Faso health-districts from 2013 to 2018

High season, period between July and November of each year. Low season, period between outside the interval of July and November of each year.

Source : The shapefile is obtained from Base Nationale de Découpage du territoire du Burkina Faso (BNDT, 2006). Maps created by Toussaint Rouamba, 2019

**
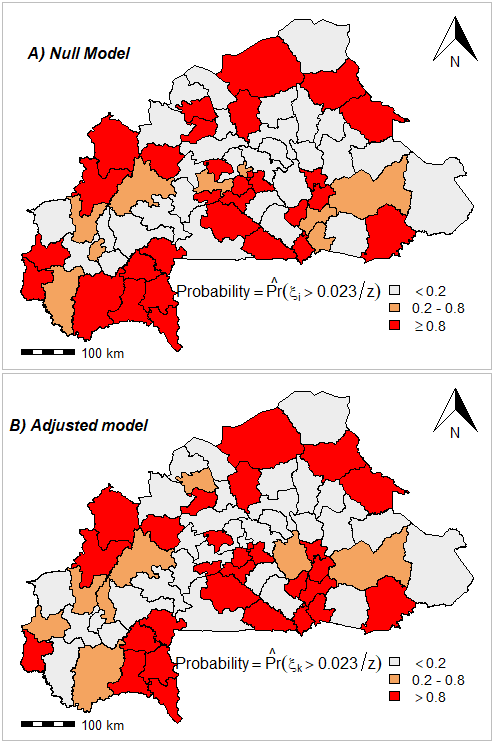
**

**Figure S6.** Burkina Faso health-districts severe-malaria cases among pregnant women: exceedance probability ($\hat{Pr}(\xi_{k}>c/z$) of posterior expected relative risk based on Richardson’s classification

c=0.19 (NMCP threshold in 2018). The threshold of 23 per 1000 pregnant women is derived from the NMCP strategy plan that aims to reduce the global annual average rate of mortality rate due to malaria by 270 per 1000 for the period 2015 to 2020.^7^ The level of SMFR in pregnant women was estimated at 450 per 1000 2015.

**
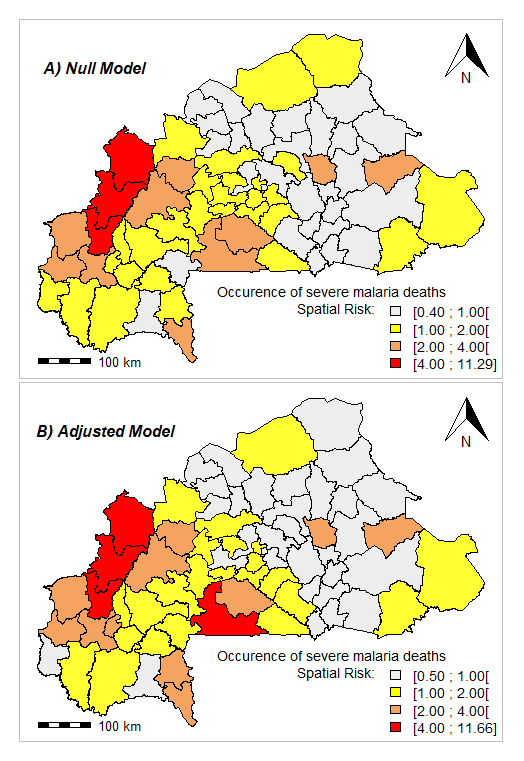
**

**Figure S7.** Posterior mean for the health-district-specific relative risks of severe-malaria deaths, $exp(\xi_{k})$

**
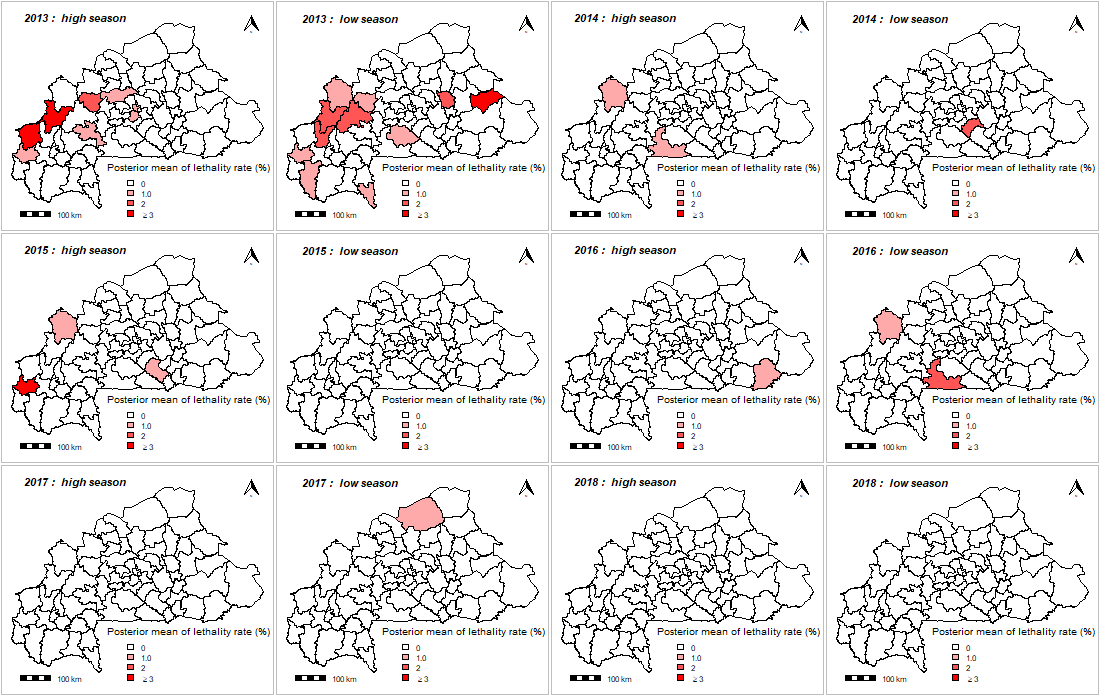
**

**Figure S8.** Geographic distribution of posterior mean of monthly deaths due to severe-malaria among pregnant women in Burkina Faso health-districts from 2013 to 2018

High season, period between July and November of each year. Low season, period between outside the interval of July and November of each year.

Source : The shapefile is obtained from Base Nationale de Découpage du territoire du Burkina Faso (BNDT, 2006). Maps created by Toussaint Rouamba, 2019
